# The continuous race of therapy optimization in sepsis control

**DOI:** 10.1186/1471-2334-14-S7-O31

**Published:** 2014-10-15

**Authors:** Doina Iovănescu, Cleo Roşculeț, Andrei Rogoz, Marius Radu, Raluca Zlotea, Cătălin Apostolescu

**Affiliations:** 1 National Institute for Infectious Diseases "Prof. Dr. Matei Balş", Bucharest, Romania

## Background

Statistical data resulted from the analysis of the cases diagnosed and treated in the ICU of INBI Matei Balş in the last 5 years. The continuous escalation of the technical means of advanced life support associated to the antibacterial and antifungal therapy required by the proven or presumed etiology.

## Methods

We analyzed risk factors, comorbidities, previous maintenance treatment schemes, a complete picture of maximal complexity which requires interdisciplinary teams.

## Results

Statistical analysis of the data from the last 10 months showed a total of 186 cases of sepsis, out of which 86 cases of severe sepsis and septic shock with a very high rate of mortality (76 patients). Risk factors and comorbidities (a high rate of obesity, cardiac diseases, diabetes and immunodeficiency, elder patients (63.6 years old), and so on) with etiology ranging from MDR GNB (*P. aeruginosa*, *A. baumannii*, *K. pneumoniae*, *Enterobacter* spp) to gram positive cocci, *C. difficile*, fungal infections, and various viral infections (Influenza v., Parainfluenza, Enteroviruses and even Hantavirus) could explain the difficulties in the management of critically ill patients.

## Conclusion

The comparative analysis with the previous years highlights the difficulties in the strategy of patient care in the ICU. Under the prescribed medication schemes there were both successes and failures, often due to the antibiotic resistance profile. The ICU cases are more and more complex, requiring the continuous optimization of the therapy schemes, advanced ICU technology, and the presence of a permanent multidisciplinary team, in the hope of achieving a better severe sepsis control.

